# Effect of Yoga on the Quality of Life of Nurses Working in Intensive Care Units. Randomized Controlled Clinical Trial

**DOI:** 10.17533/udea.iee.v37n3e06

**Published:** 2019-10-23

**Authors:** Khatereh Rostami, Fariba Ghodsbin

**Affiliations:** 1 Ph.D. Assistant professor, Shiraz University of Medical Sciences, Shiraz, Iran. Email: khaterehrostami61@yahoo.com Shiraz University of Medical Sciences Shiraz Iran khaterehrostami61@yahoo.com; 2 Ph.D. Assistant professor, Shiraz University of Medical Sciences, Shiraz, Iran. Email: ghodsbin@sums.ac.ir. Corresponding author Shiraz University of Medical Sciences Shiraz Iran ghodsbin@sums.ac.ir

**Keywords:** yoga, exercise, meditation, nurses, quality of life, intensive care units, randomized controlled trial, surveys and questionnaires, encuestas y cuestionarios., yoga, ejercicio, meditación, enfermeras y enfermeros, calidad de vida, unidades de cuidados intensivos ensayo clínico controlado aleatorio, inquéritos e questionários., ioga, exercício, meditação, enfermeiras e enfermeiros, qualidade de vida, unidades de terapia intensiva, ensaio clínico controlado aleatório.

## Abstract

**Objective.:**

The work, herein, sought to determine the effect of yoga on the quality of life of nurses working in intensive care units (ICU).

**Methods.:**

This was a randomized controlled clinical trial of a preventive intervention of three weekly sessions of yoga exercises, which included aspects of meditation, breathing control, and slow body movements. The study selected 70 nurses working in ICU and assigned them to two groups: experimental (*n* = 35) and control (*n* = 35). The World Health Organization Quality of Life brief questionnaire (WHOQoL-Bref) was used to evaluate on four moments (baseline, one, two, six months after the start of the study); this scale has 26 items with Likert-type response options ranging from 1 to 5; higher total score indicates better quality of life.

**Results.:**

The baseline score of quality of life in the experimental group was 62.3, which increased to 70.7 on the first month and continued improving in the evaluations on the second month (72.8) and sixth month (74.1), with this change being statistically significant. Instead, the control group showed no differences in scores of the different moments of evaluation (baseline = 62, first month = 61.9, second month = 62.4, and sixth month = 60.4). In the four domains of the WHOQoL-Bref (physical, psychological, social relationships, and environment), it was also noted that the experimental group obtained better scores over time compared with the control group.

**Conclusion.:**

The intervention of yoga exercises was effective in improving the quality of life of nurses working in ICU.

## Resumen

**Objetivo.:**

Determinar el efecto del yoga en la calidad de vida de las enfermeras que trabajan en Unidades de Cuidados Intensivos (UCI).

**Métodos.:**

Ensayo clínico controlado aleatorizado de una intervención preventiva de 3 sesiones semanales de ejercicios de yoga que incluyeron aspectos de meditación, control de la respiración, y movimientos corporales lentos. Se seleccionaron 70 enfermeras que trabajan en UCI y se asignaron aleatoriamente a dos grupos: experimental (*n*=35) y de control (*n*=35). El cuestionario de Calidad de Vida de la Organización Mundial de la Salud -WhoQol-Bref- se utilizó para la evaluación en cuatro momentos (basal, uno, dos y seis meses posinicio del estudio), esta escala tiene 26 items con opciones de respuesta tipo Likert de 1 a 5, a mayor puntaje total se considera que es mejor la calidad de vida.

**Resultados.:**

La puntuación basal de calidad de vida en el grupo experimental fue de 62.3, la cual se incrementó a 70.7 en el primer mes y continuó mejorando en las evaluaciones del 2º mes (72.8) y 6º mes (74.1), cambio estadísticamente significativo. Por el contrario, en el grupo control no se encontraron diferencias en el puntaje en los diferentes momentos de evaluación (basal =62, primer mes =61.9, segundo mes =62.4 y sexto mes =60.4). En los cuatro dominios de WhoQol-Bref (físico, psicológico, relaciones sociales y ambiente), también se apreció que el grupo experimental obtuvo mejores puntajes en el tiempo comparado con el grupo control.

**Conclusión.:**

La intervención con ejercicios de yoga mejoró la calidad de vida de las enfermeras que trabajan en UCI.

## Resumo

**Objetivo.:**

Determinar o efeito da yoga na qualidade de vida das enfermeiras que trabalham em Unidades de Tratamentos Intensivos (UTI).

**Métodos.:**

Ensaio clínico controlado aleatorizado de uma intervenção preventiva de 3 sessões semanais de exercícios de yoga, que incluíam aspectos de meditação, controle da respiração, e movimentos corporais lentos. Se selecionaram 70 enfermeiras que trabalham em UTI e se designaram aleatoriamente a dois grupos experimentais (*n*=35) e de controle (*n*=35). O questionário de Qualidade de Vida da Organização Mundial da Saúde -WhoQol-Bref- se utilizou para a avaliação em quatro momentos (basal, um, dois e seis meses pós início do estudo), esta escala tem 26 itens com opções de resposta tipo Likert de 1 a 5, a maior pontuação total se considera que é melhor a qualidade de vida.

**Resultados.:**

A pontuação basal de qualidade de vida no grupo experimental foi de 62.3, a qual se incrementou a 70.7 no primeiro mês e continuou melhorando nas avaliações do 2º mês (72.8) e 6º mês (74.1), sendo este câmbio estatisticamente significativo. Em câmbio, no grupo controle não se encontraram diferenças na pontuação nos diferentes momentos de avaliação (basal =62, primeiro mês =61.9, segundo mês =62.4 e sexto mês =60.4). Nos quatro domínios de WhoQol-Bref (físico, psicológico, relações sociais e ambiente), também se apreciou que o grupo experimental obteve melhores pontuações no tempo comparado com o grupo de controle.

**Conclusão.:**

A intervenção de exercícios de yoga foi efetiva no melhoramento da qualidade de vida das enfermeiras que trabalham nas UTIs.

## Introduction

From the world health point of view, the concept of quality of life is influenced by physical health, psychiatric manners, independency level, personality development, social relationships, and interaction with outstanding organizations in the environment. quality of life includes a range of mental and objective dimensions which interact with one another; hence, living a high quality of life leads to a positive feeling for individuals. quality of life can be described in terms of person’s complete health welfare and consists of different dimensions like physical, intellectual, psychological, role performance, and social support welfare.(1) 

 Researchers believe that investigating quality of life and endeavoring for its improvement plays a great role in person’s health and their social and individual lives.(2) Various factors like age, diseases, and social environment may have positive and negative effects on the quality of life. Meanwhile, one of the effective factors is job stresses.(3) Stress is a main problem in every society that involves individuals physically and mentally.(4) Although every occupation has its own sources of stress, in jobs encountering with people’s health, this issue is of a higher importance. Medical team members, particularly, nurses are among the people who experience higher levels of professional stress.(2) Thus, their health and quality of life is strongly influenced by stress and risk of diseases like diabetes, cardiovascular, and other chronic diseases.(5) In addition, stress directly affects quality of life, job satisfaction, professional exhaustion, increase in medical faults, and therefore decreasing the quality of healthcare services for patients.(6) Nurses working in intensive care unit, due to conditions of their job, physical environment of the ward, type of patients, and their tense conditions, as well as their heavy duties experience more stresses.(7,8) 

There are different strategies to control occupational stresses and therefore, improving the quality of life.(9,10) Matsumoto has divided the strategies of coping with stress into three groups of emotion focus, problem focus, and ineffectiveness.(11) In the emotion focus strategy, person does not make any effort to decrease and control stress, but he tries to calm himself and get rid of stress. In the problem focus strategy, the individual tries to decrease or control his occupational stress. Unfortunately, the majority of people use the ineffectiveness strategy. In this strategy the individuals neither solves the problem nor decreases it; he usually uses inefficient harmful strategies that cause negative outcomes to control stress.(12) Yoga is one of these strategies which has a significant role in controlling stress.(13) Yoga, actually is a set of movements that coordinate body and soul and create flexibility against negative pressures and increase ability to cope with stress.(14) Yoga has positive short-term and long-term effects on physical stress that consequently improves the quality of life.(15) Even though it has been tried to teach treatment to yoga team through different studies, there is not any study done directly to investigate the effects of yoga on the quality of life of nurses working in intensive care units (ICU).(16) Then, the researchers decided to investigate the effect of this strategy on the quality of nurses’ lives working in ICU. 

## Methods

This clinical trial study was conducted in 2017. According to previous studies the sample size was 70 nurses working in intensive care units. After the approval of the study by the ethics committee of Shiraz University of Medical Sciences, the study was started. The eligibility criteria for participants were items like not using painkillers, having at least six months of job experience, and tendency to participate in the study. The criteria for exiting the study included participating in any of aerobics classes, meditation, and unwillingness to continue their cooperation with the research. In order to determine the quality of nurses lives, quality of life Who-QOL-Bref questionnaire was used whose reliability and validity have been admitted by Sharbaf *et al.*(17) This questionnaire consists of 26 questions. Grading is defined as one point for *at all* choices*,* five points for *very much* choices, and for questions 3, 4, and 26 which have negative connotations, it is contrariwise. Higher points represent higher quality of life. For conforming this questionnaire, grading to WHO 100-question complete questionnaire, each of the points will be multiplied by 4 in order to gain the main grade. Settings and locations where the data were collected were special sections of all Shiraz Medical Sciences Hospitals. Qualified people who were willing to participate in this research were registered until the list reached 70 people. The selected nurses were divided randomly into two equal case and control groups by using Simple Block Random sampling method. 

In the introduction meeting, after receiving letters of satisfaction and explaining that entering and exiting this research is optional, first the nurse’s quality of life was investigated in four physical (6 questions), psychological (7 questions), social relations (3 questions), and environmental (8 questions) before starting the classes. Then, the interference group participated in yoga classes for two months and twice a week. One, two, and six months after the classes were finished; the questionnaire completed by both interference and control groups and then, gathered.

In each session of yoga classes, three aspects of mind control, breathe control, and slow body movements were worked out. Yoga movements start as the participants lying down on their backs during which they practice concentrating on mind and breathe for fifteen minutes. In this condition, the individual’s mind is turned rotatory in different parts and it is attempted to teach the individual how to control his mind. After that, and in this condition, four slow body movements are exercised. After doing each movement, the patient’s mind is concentrated on the intended organ so that he can have a better perception of it after concentration. After each movement, the breathing and mind control practice was done on that organ. Afterwards, three slow movements are done in sitting condition and after each movement, psychological and mental attention is paid to that organ. Next, the person does four slow movements as he stands and after each movement, his psychological and mental attention is paid to the intended organ; at the end, the breath control is done. After that, the person is set in lying position again and his mind is being worked out on for fifteen minutes until he reaches a good feeling towards himself and the world around him. 

For ethical considerations through the research process from start to the end, the researcher emphasized conscious satisfaction, nurses’ anonymity, confidentiality of the information, the right to leave the participation at any optional time.

**Figure 1:**
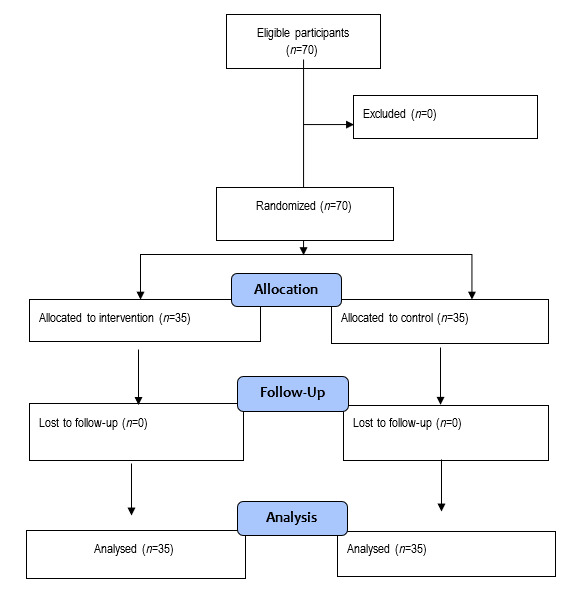
Flow Diagram of the study

## Results

The average age of the experimental group is 30.5±5.14 years versus 29.3±5.1 years in the control group. The average of experience (in a year) for experimental group is 6.23±3.1 and in the control group, it is 5.9±2.8. The average of work time in the week for experimental group is 40.55 ± 2.2 and in control group, it is 38.41 ± 4.3. The amount of monthly salary for experimental group (in Tomans) is 1834600±61500 and in control group, it is 1822540 ± 51000. It has been determined that there is no meaningful statistical difference between experimental and control groups in terms of quantitative demographic information. Also, there is no meaningful statistical difference between the two experimental and control groups in terms of gender, marital status, number of children, shift conditions, and job satisfaction. 

The results of how yoga classes influence the quality of intensive care unit nurses’ lives are illustrated in Table 1. The global score of nurses’ life qualities between groups before intervention has no meaningful statistical difference.

**Table 1:** Comparison of the intervention and control groups regarding the global and dimensions mean scores of WhoQOL - Bref by time

		Time	Time	Time		
Dimension	Group					Time by group *p*-value
		Before	One month	Two months	Six months	
Physical	Experimental	61.02±2.03	68.3±3.1	70.14±3.1	75.1±2.15	<0.001
	Control	62.3±2.9	61.1±3.04	62.1±2.3	59.3±1.52	
	*Between group p-value*	*<0.05*	*<0.001*	*<0.001*	*<0.001*	
Psychological	Experimental	62.2±3.1	70.01±2.6	73.3±3.0	73.2±1.88	<0.001
	Control	60.3±3.4	61.8±2.4	62.2±1.8	58.8±1.96	
	*Between group p-value*	*<0.05*	*<0.001*	*<0.001*	*<0.001*	
Social Relations	Experimental	63.01±2.2	71.2±2.9	72.6±2.8	73.8±1.95	<0.001
	Control	64.3±1.5	63.9±2.5	64.2±2.4	61.9±2.3	
	*Between group p-value*	*<0.05*	*<0.001*	*<0.001*	*<0.001*	
Environment	Experimental	62.9±2.05	73.04±2.1	75.5±2.4	74.05±2.00	<0.001
	Control	61.1±2.04	60.9±3.3	61.3±2.5	60.1±1.92	
	*Between group p-value*	*<0.05*	*<0.001*	*<0.001*	*<0.001*	
Global	Experimental	62.2±2.38	70.72±2.6	72.8±2.8	74.1±2.03	<0.001
	Control	62±2.46	61.9±2.8	62.4±2.2	60.4±1.9	
	*Between group p-value*	*<0.05*	*<0.001*	*<0.001*	*<0.001*	

As it is obvious, the average total score of nurses’ quality of life in experimental group has increased meaningfully in 1 month and 2 months after interference comparing to that of prior intervention in the four dimensions of WHOQOL-BREF. Therefore, time was an effective factor for dimensions of this change, with respect to P-V which has become meaningful, it can be said that the changes process in experimental group comparing with this process in control group have a meaningful difference. 

## Discussion

There are several factors in every job that influence the job holder’s quality of life. In the present study, the process of changes in nurses’ general quality of life who work in intensive care units and in the time span of six months and earlier to the end of interferences showed the effectiveness of yoga exercises on the quality of their lives. Since there were not any significant differences between the two groups’ biological variables, it can be said that changes in the general quality of service users’ lives of yoga group were resulted from the interferences made more confidently. In similar researches also, the quality of different groups’ lives has improved significantly by applying yoga interferences.(18,19) Lin(19) set to investigate the effect of 12 weeks of yoga exercises on controlling factors affecting quality of life like stress and strategies of coping with stress in mental health professionals that consequently showed positive effect of yoga training on these factors.

In this study, the comparison of two groups’ averages before starting the interferences revealed that the condition of control group, in terms of general quality of life and its realms comparing to interference group, does not have any meaningful statistical differences. After eight weeks of training, total grade of quality of life and its realms in yoga group increased when compared to control group. In this investigation, and according to the findings, in four physical, psychological, social relations, and environment subscales, there has been a meaningful improvement in yoga group; and also there were more changes in this group than in the control group. This represents effectiveness of yoga program in augmenting nurses’ quality of life. Although quality of life grade in the first month after trainings has improved remarkably, the changes in this group through two and six months later was not that much noteworthy. Considering that the yoga program for nurses was short-term, it seems that the reason lies in these trainings, not being long-lasting during their workdays. 

In this study, meaningful statistical differences were seen between the two yoga and control groups in terms of physical, psychological, social relations, and environment subscales. A study by Tekur *et al.*(20) also admits this after a one month period of yoga exercises with the purpose of investigating the effect of yoga on physical, mental, emotional and intellectual dimensions and four physical, psychological, social relations, and environment subscales on the quality of life of patients suffering from chronic backache, a meaningful improvement was seen before and after interference. The findings of paired t-test in the study conducted by McDermott *et al.*(21) showed that after yoga trainings, the grade of quality of life in physical performance, physical role, general health status, and psychological role dimensions have had a great meaningful increase which corresponds with the findings of the present study. 

In the study by Jyotsna *et al.*,(22) also yoga exercise were practiced with 49 patients suffering diabetes type 2 for six months, and results revealed that the average of body performance and role dimensions grew about 3 points that is statistically meaningful. Yoga can lead to increase in quality of life and the feeling of health in the whole body by adjusting automatic psychological, neural, immunity systems, enhancing body stability and resistance, and regulating immunity system.(23) Yoga exercises, in this study, resulted in improvements in physical and body dimensions. Because yoga body and breathing exercises enhances flexibility and muscular strength, improves circulation, oxygen absorption, and hormone system performance, and finally, it can boost both body performance and body role.(24) Evaluating the studies show that medical interferences concentrating on activity level (like yoga and progressive-resistive exercise) will have more positive effects on patients’ abilities to do their daily works, and consequently on their quality of life.(25)

In the present study, a meaningful increase was seen in the average grades of psychological health and psychological role in yoga group comparing to control group. In the study by Jyotsna also, an increase in the average of two psychological health and psychological role after practicing the exercise has been reported.(22) These results adjust with that of other studies.(21,26). Due to the outstanding effect that yoga has in relaxing, people enjoy an endless calmness. Yoga is a set of exercises and body stretches that are accompanied by releasing psychological energies exercises. It emphasizes on the principle of balance between psychological and body powers as well as decreasing stress.(13) Therefore, it seems reasonable that after a period of yoga exercise, nurses’ psychological health grade boosts. In this study, the effects of yoga on improving health, social relations, and environment’s safety were positive and meaningful in a way that the grade of these two dimensions of quality of life in experimental group improved remarkably before interference up to six months after that. So, this finding adjusts with that of some researches so that it is said trainings led to this boost in social and environmental health dimensions.(27) Accordingly, yoga can be used as an effective, convenient, and inexpensive method to improve quality of life of these people.

## Conclusion.

The results of this study illustrated that yoga improve the quality of life of nurses working in Intensive Care Units. Considering the fact that nurses’ job stresses grow every day and therefore, the expenses causing from them increase too, and also with respect to the advantages of yoga exercise like not containing any side effects, inexpensiveness, easy to do, availability, not being offensive, and applicability of exercise, it is very valuable, and, nurses can use yoga as an extracurricular activity. 

Among the limitations of this research, we can name the selection of participants from the available and people who volunteered to take part in classes; so it cannot be considered as the representative of the whole society. This confines generalizing the results of this research to all nurses who work in intensive care units. It is suggested that appropriate yoga exercises be practiced in order to increase coping with job stress at home and medical centers by nurses so that continuing these exercises endures the effect of these exercises.
